# Genome Sequence of Bordetella pertussis Vaccine Strain BP 165

**DOI:** 10.1128/MRA.00150-19

**Published:** 2019-04-18

**Authors:** Shweta Alai, Vikas C. Ghattargi, Manish Gautam, Dhiraj P. Dhotre, Krunal Patel, Shrikant P. Pawar, Rakesh Kumar, Umesh Shaligram, Deepti Deobagkar, Sunil Gairola

**Affiliations:** a Department of Health and Biological Sciences, Symbiosis International University, Pune, Maharashtra, India; b Serum Institute of India Private Limited, Pune, Maharashtra, India; c National Centre for Microbial Resource, National Centre for Cell Science, Pune, Maharashtra, India; d ISRO Space and Technology Cell and Molecular Research Laboratory, Department of Zoology, Savitribai Phule Pune University, Pune, Maharashtra, India; Georgia Institute of Technology

## Abstract

Whole-cell and acellular pertussis vaccines are used globally against Bordetella pertussis. Various vaccine reference strains are used globally for the production of such vaccines. We report here a draft genome sequence for Bordetella pertussis strain BP 165, which is used by the Serum Institute of India in the production of acellular pertussis vaccine.

## Abstract (precis)

Whole-cell and acellular pertussis vaccines are used globally against Bordetella pertussis. Various vaccine reference strains are used globally for the production of such vaccines.

## ANNOUNCEMENT

Bordetella pertussis, a Gram-negative bacterium, is the causative agent of pertussis (whooping cough) in humans (1). Despite high vaccination coverage, resurgence of pertussis disease has been reported in many countries. The observed resurgence of whooping cough underlines the need for studies on strain evolution, antigenic divergence at local and global levels, and vaccine-induced adaptation in the pathogen (2). We report here a draft genome sequence of B. pertussis strain BP 165, which is used in the manufacture of acellular pertussis vaccine at the Serum Institute of India. Bordetella pertussis strain BP 165 is a U.S. clinical isolate procured from the Center for Biologics Evaluation and Research (CBER) in the United States. The strain was grown on Bordet-Gengou agar slants supplemented with 15% horse blood and modified Stainer Scholte (SS) medium incubated at 35°C for 3 days.

Genomic DNA was extracted and purified using a genomic DNA Clean and Concentrator-10 kit (Zymo Research) per the manufacturer’s instructions. Whole-genome sequencing was performed using the MiSeq platform (Illumina, San Diego, CA). The MiSeq shotgun library was prepared using an NEBNext Ultra DNA library prep kit and was analyzed with a Bioanalyzer instrument (Agilent Technologies), using a DNA 1000 chip per the manufacturer’s instructions, followed by 2 × 150-bp paired-end sequencing. The 1,245,512 paired-end reads generated were quality filtered with 87-fold mean coverage. Adapter and low-tone sequences were removed using Trimmomatic v0. 35 (3) with a sliding window quality cutoff of Q20. *De novo* assembly was performed using SPAdes v3.7 (4). A total of 1,186,989 reads were assembled into 264 contigs with an *N*_50_ value of 68,043 bp. Genome annotation was performed using the NCBI Prokaryotic Genome Automatic Annotation Pipeline, PATRIC (5), and Rapid Annotations using Subsystems Technology (RAST) tools (6). CRISPR repeats and bacteriophages were predicted using CRISPR Finder (7) and PHAge Search Tool Enhanced Release (PHASTER) tools (8), respectively. Bacterial insertion elements (ISs) were identified by ISfinder (9), and horizontal gene transfer was detected by the genomic island tool IslandViewer (10). All of the programs and tools in the study were used with default settings.

The genome of *B. pertussis* vaccine strain BP 165 consists of 4,101,762 bp, with a mean GC content of 67.71%. Totals of 3,702 genes, 46 structural tRNAs, 3 rRNAs, and 4 noncoding RNAs (ncRNAs) were predicted. The genome analysis suggested the absence of plasmids, phages, and CRISPRs. Multilocus sequence typing (MLST) analysis demonstrated that B. pertussis strain BP 165 belongs to clonal complex II (sequence type 2 [ST2]) (11). Core genome multilocus sequence typing showed its similarity with global allelic profile 41 (12). The virulence factors were also identified as being *prn* (1), *ptxA* (2), *ptxC* (2), *ptxP* (1), *fim2* (1), *fim3* (1), *tcfA* (2), *ompQ* (2), and *vag8* (2) alleles. Insertion sequences IS*481*, IS*1663*, and IS*1002* in strain BP 165 were compared with those in the *B*. *pertussis* reference strain Tohama-I and *B. pertussis* CS and were found to be similar (13).

The accessibility of high-quality genome sequences of B. pertussis vaccine strains will allow detailed phylogenetic and bioinformatic studies, which will facilitate better understanding of sequence variations globally, thus opening the way for global surveillance.

### Data availability.

This whole-genome shotgun project has been deposited at GenBank under accession number RSFF00000000, BioProject number PRJNA508597, BioSample number SAMN10525643, and Sequence Read Archive accession number SRX5485178. The version of the project described here is version number RSFF01000000, and it consists of sequences RSFF01000001 to RSFF01000264.

## References

[1] Domenech de Cellès M, Magpantay FMG, King AA, Rohani P 2016 The pertussis enigma: reconciling epidemiology, immunology and evolution. Proc R Soc B 283:20152309. doi:10.1098/rspb.2015.2309. PMC4721090 26763701

[2] He Q, Mertsola J 2008 Factors contributing to pertussis resurgence. Future Microbiol 3:329–339. doi:10.2217/17460913.3.3.329. 18505398

[3] Bolger AM, Lohse M, Usadel B 2014 Trimmomatic: a flexible trimmer for Illumina sequence data. Bioinformatics 30:2114–2120. doi:10.1093/bioinformatics/btu170. 24695404 PMC4103590

[4] Bankevich A, Nurk S, Antipov D, Gurevich AA, Dvorkin M, Kulikov AS, Lesin VM, Nikolenko SI, Pham S, Prjibelski AD, Pyshkin AV, Sirotkin AV, Vyahhi N, Tesler G, Alekseyev MA, Pevzner PA 2012 SPAdes: a new genome assembly algorithm and its applications to single-cell sequencing. J Comput Biol 19:455–477. doi:10.1089/cmb.2012.0021. 22506599 PMC3342519

[5] Wattam AR, Davis JJ, Assaf R, Boisvert S, Brettin T, Bun C, Conrad N, Dietrich EM, Disz T, Gabbard JL, Gerdes S, Henry CS, Kenyon RW, Machi D, Mao C, Nordberg EK, Olsen GJ, Murphy-Olson DE, Olson R, Overbeek R, Parrello B, Pusch GD, Shukla M, Vonstein V, Warren A, Xia F, Yoo H, Stevens RL 2017 Improvements to PATRIC, the all-bacterial bioinformatics database and analysis resource center. Nucleic Acids Res 45:D535–D542. doi:10.1093/nar/gkw1017. 27899627 PMC5210524

[6] Aziz RK, Bartels D, Best AA, DeJongh M, Disz T, Edwards RA, Formsma K, Gerdes S, Glass EM, Kubal M, Meyer F, Olsen GJ, Olson R, Osterman AL, Overbeek RA, McNeil LK, Paarmann D, Paczian T, Parrello B, Pusch GD, Reich C, Stevens R, Vassieva O, Vonstein V, Wilke A, Zagnitko O 2008 The RAST server: Rapid Annotations using Subsystems Technology. BMC Genomics 9:75. doi:10.1186/1471-2164-9-75. 18261238 PMC2265698

[7] Grissa I, Vergnaud G, Pourcel C 2008 CRISPRcompar: a website to compare clustered regularly interspaced short palindromic repeats. Nucleic Acids Res 36:W145–W148. doi:10.1093/nar/gkn228. 18442988 PMC2447796

[8] Arndt D, Grant JR, Marcu A, Sajed T, Pon A, Liang Y, Wishart DS 2016 PHASTER: a better, faster version of the PHAST phage search tool. Nucleic Acids Res 44:W16–W21. doi:10.1093/nar/gkw387. 27141966 PMC4987931

[9] Siguier P, Perochon J, Lestrade L, Mahillon J, Chandler M 2006 ISfinder: the reference centre for bacterial insertion sequences. Nucleic Acids Res 34:D32–D36. doi:10.1093/nar/gkj014. 16381877 PMC1347377

[10] Dhillon BK, Laird MR, Shay JA, Winsor GL, Lo R, Nizam F, Pereira SK, Waglechner N, McArthur AG, Langille MGI, Brinkman SFL 2015 IslandViewer 3: more flexible, interactive genomic island discovery, visualization and analysis. Nucleic Acids Res 43:W104–W108. doi:10.1093/nar/gkv401. 25916842 PMC4489224

[11] Diavatopoulos DA, Cummings CA, Schouls LM, Brinig MM, Relman DA, Mooi FR 2005 *Bordetella pertussis*, the causative agent of whooping cough, evolved from a distinct, human-associated lineage of *B. bronchiseptica*. PLoS Pathog 1:e45. doi:10.1371/journal.ppat.0010045. 16389302 PMC1323478

[12] Bouchez V, Guglielmini J, Dazas M, Landier A, Toubiana J, Guillot S, Criscuolo A, Brisse S 2018 Genomic sequencing of *Bordetella pertussis* for epidemiology and global surveillance of whooping cough. Emerg Infect Dis 24:988–994. doi:10.3201/eid2406.171464. 29774847 PMC6004856

[13] Zhang S, Xu Y, Zhou Z, Wang S, Yang R, Wang J, Wang L 2011 Complete genome sequence of *Bordetella pertussis* CS, a Chinese pertussis vaccine strain. J Bacteriol 193:4017–4018. doi:10.1128/JB.05184-11. 21622744 PMC3147532

